# Novel mutations in the *SGCA* gene in unrelated Vietnamese patients with limb-girdle muscular dystrophies disease

**DOI:** 10.3389/fgene.2023.1248338

**Published:** 2023-10-13

**Authors:** Nam Chung Tran, Nguyen Thi Kim Lien, Thanh Dat Ta, Van Hung Nguyen, Huy Thinh Tran, Nguyen Van Tung, Nguyen Thi Xuan, Nguyen Huy Hoang, Van Khanh Tran

**Affiliations:** ^1^ Center for Gene and Protein Research, Department of Molecular Pathology, Faculty of Medical Technology, Hanoi Medical University, Hanoi, Vietnam; ^2^ Hanoi Medical University, Hanoi, Vietnam; ^3^ Institute of Genome Research, Vietnam Academy of Science and Technology, Hanoi, Vietnam; ^4^ Graduate University of Science and Technology, Vietnam Academy of Science and Technology, Hanoi, Vietnam

**Keywords:** limb-girdle muscular dystrophies (LGMDs), mutation, the *CAPN3* gene, the *SGCA* gene, Vietnamese patients

## Abstract

**Background:** Limb-girdle muscular dystrophy (LGMD) is a group of inherited neuromuscular disorders characterized by atrophy and weakness in the shoulders and hips. Over 30 subtypes have been described in five dominant (LGMD type 1 or LGMDD) and 27 recessive (LGMD type 2 or LGMDR). Each subtype involves a mutation in a single gene and has high heterogeneity in age of onset, expression, progression, and prognosis. In addition, the lack of understanding of the disease and the vague, nonspecific symptoms of LGMD subtypes make diagnosis difficult. Even as next-generation sequencing (NGS) genetic testing has become commonplace, some patients remain undiagnosed for many years.

**Methods:** To identify LGMD-associated mutations, Targeted sequencing was performed in the patients and Sanger sequencing was performed in patients and family members. The *in silico* analysis tools such as Fathmm, M-CAP, Mutation Taster, PolyPhen 2, PROVEAN, REVEL, SIFT, MaxEntScan, Spliceailookup, Human Splicing Finder, NetGene2, and Fruitfly were used to predict the influence of the novel mutations. The pathogenicity of the mutation was interpreted according to the ACMG guidelines.

**Results:** In this study, six patients from four different Vietnamese families were collected for genetic analysis at The Center for Gene and Protein Research and The Department of Molecular Pathology Faculty of Medical Technology, Hanoi Medical University, Hanoi, Vietnam. Based on clinical symptoms and serum creatine kinase (CK) levels, the patients were diagnosed with limb-girdle muscular dystrophies. Five mutations, including four (c.229C>T, p.Arg77Cys; exon one to three deletion; c.983 + 5G>C; and c.257_258insTGGCT, p.Phe88Leufs*125) in the *SGCA* gene and one (c.946-4_946-1delACAG) in the *CAPN3* gene, were detected in six LGMD patients from four unrelated Vietnamese families. Two homozygous mutations (c.983 + 5G>C and c.257_258insTGGCT) in the *SGCA* gene were novel. These mutations were identified as the cause of the disease in the patients.

**Conclusion:** Our results contribute to the general understanding of the etiology of the disease and provide the basis for definitive diagnosis and support genetic counseling and prenatal screening.

## Introduction

Limb-girdle muscular dystrophy (LGMD) is a group of neuromuscular disorders that were divided into subtypes based on the inheritance patterns (Iyadurai and Kissel, 2016). LGMD was identified to be caused by gene mutations that encode proteins presenting in the extracellular matrix, sarcolemma, cytoplasm, and nucleus (van der Kooi, 2017). More than 30 subtypes of LGMD have been identified as autosomal dominant (LGMD type 1 or LGMDD) and autosomal recessive (LGMD type 2 or LGMDR) subtypes (Supplementary Table S1) (Chu and Moran, 2018; Khadilkar et al., 2018; Straub et al., 2018; Wicklund, 2019; Angelini, 2020). The prevalence of LGMD across all subtypes ranged from 0.8 to 6 per 100,000 and the carrier ratio was from 1:150 to 1:211 (Theadom et al., 2014; Deenen et al., 2015; Chu and Moran, 2018). However, the autosomal recessive forms (with a ratio of 1:15,000) are more common than the autosomal dominant forms. Most subtypes of LGMD affect both sexes equally. Depending on the subtype, the age of onset can vary from childhood to adulthood, however, males in R3/2D and R5/2C subtypes may have an earlier onset (Magri et al., 2017). All subtypes of LGMD are characterized by weakness and atrophy of the muscles around the shoulders and hips. LGMD patients have difficulty standing up, sitting down, or climbing stairs, some patients may need a wheelchair or other assistive devices to move around. To date, although many experimental treatments are being developed, none have been able to interrpt the progression of the disease. Medical treatment for patients with LGMD mainly includes physical therapies that can help the patients in daily living or control the progression of the disease.

Patients in different subtypes and even within the same subtype have a difference in the age of onset and the progression of the disease. In general, patients with an earlier age of onset tend to have a faster progression of the disease. Patients with early-onset may rapidly develop proximal muscle weakness leading to loss of independent mobility within 10 years. These patients are more likely with cardiovascular and respiratory complications than those with late-onset. Patients with late-onset progress more slowly and may maintain the ability to walk through the third and fourth decades of life. Studies have shown that the rate of disease progression is variable among LGMD subtypes: slow progression in dysferlinopathy and telethoninopathy; moderate in calpainopathy and fukutin-related proteinopathy; and rapid in sarcoglycanopathy (Mitsuhashi and Kang, 2012). The prognosis of LGMD also depends on the subtype, age of onset, and rate of progression of the disease. Therefore, the identification of the LGMD subtypes plays a very important role in genetic counseling, predicting the risk of cardiovascular and respiratory complication and prognosis in the treatment of the disease. However, in the Caucasian population, patients with LGMDR5/2C and LGMDR6/2F usually present a Duchenne-like phenotype and those with LGMDR3/2D and LGMDR4/2E have a Becker-like phenotype (Alonso-Pérez et al., 2020). The overlapping clinical manifestations among LGMD subtypes and other neuromuscular disorders (NMDs) make diagnosis difficult (Nigro and Savarese, 2014; Fanin and Angelini, 2016; Okazaki et al., 2020; Hsu et al., 2021). In addition, the general lack of awareness about LGMD and the nonspecificity of symptoms creates a challenge in definitive diagnosis. Even as next-generation sequencing (NGS) genetic testing has become commonplace, some patients remain undiagnosed (Kadoya et al., 2017; Younus et al., 2019; Li et al., 2020; Martens et al., 2021).

In the early stages of the disease, patients with LGMD often have a high serum creatine kinase (CK) level due to the release of damaged muscle cells, so this CK level can use as a criterion in the diagnosis for LGMD. However, CK levels can change during muscle activity or decline over the progression of the disease (Zhu et al., 2015). Besides that, electromyography (EMG) can be used for the differential diagnosis of muscular or neurological disorders (Narayanaswami et al., 2014). In recent years, magnetic resonance imaging (MRI) has proven to be an effective diagnostic tool in detecting and quantifying forms of muscle degeneration (Arrigoni et al., 2018). Before genetic testing became widely used, muscle biopsies and immunohistochemical staining were considered the methods to provide the final biochemical evidence to confirm and classify LGMD subtypes. However, muscle biopsies lack specificity, so genetic testing is increasingly widely used thanks to improvements and reduced cost in recent years (Wicklund, 2019). Therefore, genetic analysis has been used in the confirmatory diagnosis in approximately 75% of the LGMD patients.

The *SGCA* gene encodes for one of the fours subunits of the sarcoglycan protein complex (including α, β, γ, and δ-sarcoglycans (SG) encoded by the *SGCA*, *SGCB*, *SGCG*, and *SGCD* genes, respectively). Sarcoglycan proteins located in the membrane of muscle cells play mechanical and nonmechanical roles in stabilizing plasma membrane in cardiac and skeletal muscle. In addition, the sarcoglycan complex is bound to and acts as a structure connecting the extracellular and intracellular matrix that assists during muscle contraction to stabilize the dystrophin complex (Gao and McNally, 2015). Sarcoglycan complex identified to be involved in changes in MAPK pathway phosphorylation and mechanosensitivity in skeletal muscle cells (Barton et al., 2020). Mutations occurring in subunits of the sarcoglycan complex lead to tissue damage and cause the LGMDR3/2D - LGMDR6/2F subtypes (Al-Zaidy et al., 2015). Disease severity varies with the age of onset, rate of progression, and genotype of the patients. It has been found that patients carrying nonsense mutations exhibit the most severe phenotype and later onset patients have a better prognosis (Iyadurai and Kissel, 2016). The severity of the disease is inversely proportional to the amount of α-sarcoglycan protein in the muscle cells (Xie et al., 2019). The amounts of α-sarcoglycan protein can vary from mildly reduced to complete absence in patients with LGMDR3/2D.

Besides, mutations in the calpain-3 (*CAPN3*) gene have been identified as the cause of the LGMDR1/2A subtype (Richard et al., 1995; Kramerova et al., 2012; 2018). LGMDR1/2A is considered the most common subtype and the ratio of gene carriers in the population is 1:100. Some patients have an abnormal gait, mobility difficulties when climbing stairs, and carrying heavy objects due to muscle weakness in both lower limbs and the whole body (Straub et al., 2018). The *CAPN3* gene, located on chromosome 15q15.1, encodes a member of the Ca^2+^-activated neutral protease family that is specific to the muscle and plays an important role in muscle remodeling (Chen et al., 2021). The structure of the CAPN3 protein contains three insertion sequences, namely, the N-terminal segment (NS), insertion sequence 1 (IS1) on PC2, and insertion sequence 2 (IS2) located between CBSW/C2L and PEF (L) (McCartney et al., 2018). The IS1 domain is responsible for the activation of substrate binding during cleavage; the CBSW domain participates in CAPN3 truncation through the PEF domain; the PEF domain includes four Ca^2+^ binding sites 1, 2, 3, and 5 on EF-hand that contribute to the promotion of CAPN3 homodimerization (Ye et al., 2018). In skeletal muscle fibers, the CANP3 enzyme plays an important role in controlling Ca2+ level is essential to ensure the normal function of skeletal muscle (Ojima et al., 2011; Gehlert et al., 2015). CAPN3 can also bind to proteins involved in the control of cell signaling and muscle fiber elasticity (Kopanos et al., 2019). Therefore, mutations that leading to the loss of function of the CAPN3 enzyme were identified as the cause of muscular dystrophy (LGMDR1/2A subtype) (Richard et al., 1995).

In this study, Targeted sequencing was performed to identify mutations in Vietnamese patients with LGMD. Information about mutation patterns in patients will help doctors have the basics for definitive diagnosis, treatment, and genetic counseling for patients.

## Materials and methods

### Patients and clinical information

**FIGURE 1:**
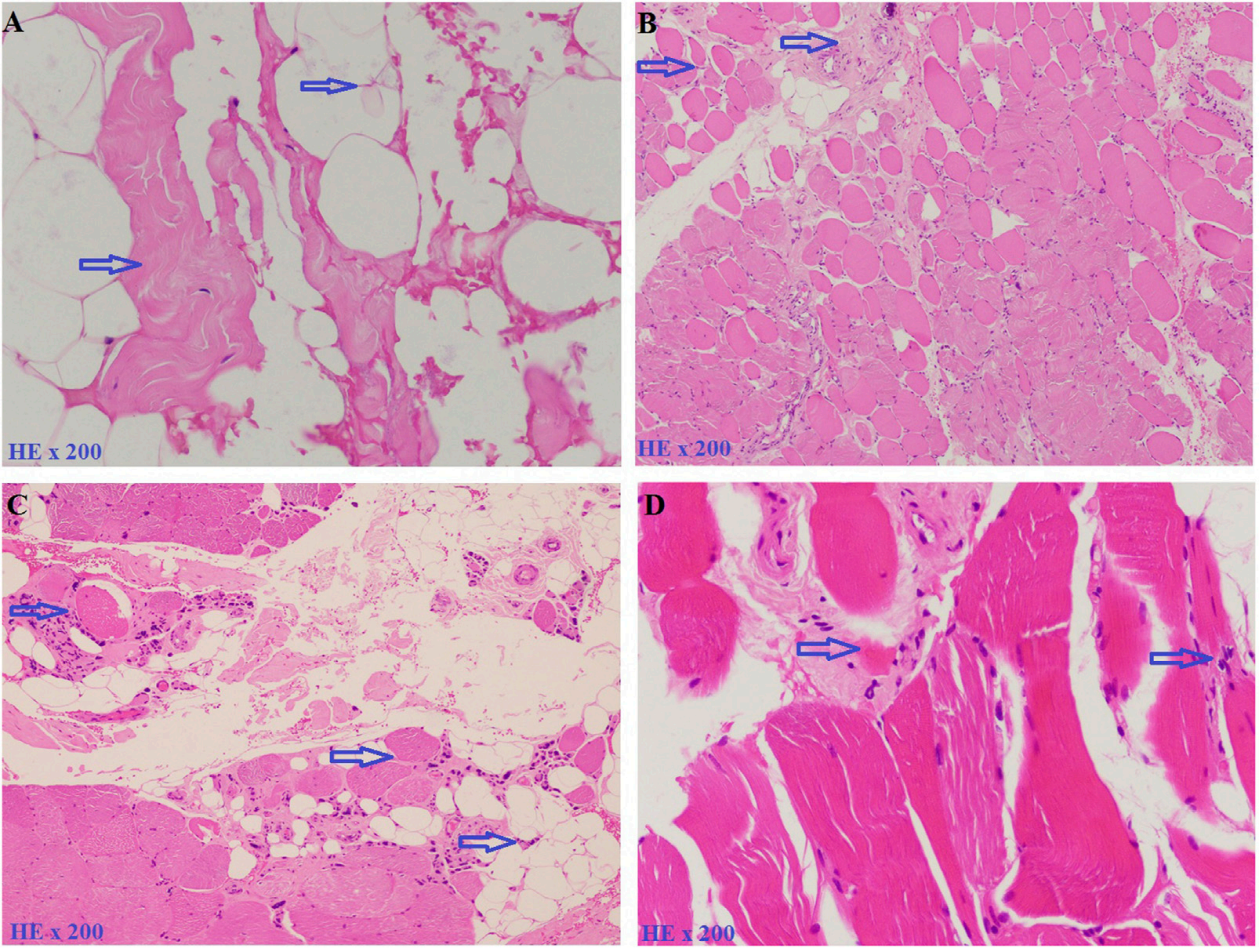
Muscle biopsies were analyzed by hematoxylin and eosin (H&E) stains. **(A)**. Patient F1-II1, including *an* increase of connective and adipose tissue (blue arrow). **(B)**. Patient F1-II2, including fiber size variation and endomysial fibrofatty degeneration (blue arrow). **(C)**. Patient F2-II1, including fiber size variation, internal nuclei, and *an* increase of connective and adipose tissue (blue arrow). **(D)**. Patient F2-II3, including fiber size variation and endomysial fibrofatty degeneration (blue arrow).

Patients from four unrelated families were collected at The Center for Gene and Protein Research and The Department of Molecular Pathology Faculty of Medical Technology, Hanoi Medical University, Hanoi, Vietnam. The serum creatine kinase (CK) levels were tested for the diagnosis of LGMD, and patients’ detailed clinical information is listed in Table 1. Family 1 (F1) has three children including two girls and one boy, with a daughter and a son who have clinical manifestations of LGMD. Patient F1-II1 is a female, onset at the age of 7 with clinical symptoms of muscle weakness in the shoulder and arms, that lead to difficulty standing up and sitting down. A year later, she showed a lumbar lordosis symptom. The patient’s brother (patient F1-II2) had the onset at the age of 8 with similar clinical symptoms and a high CK level of 16,408.3 U/L (the normal range is 22–198 U/L). However, patient F1-II2 did not have lumbar lordosis symptoms. The younger sister in the family, a 5 years-old girl has no symptoms. Family 2 (F2) has three children: two girls and one boy. The first daughter (patient F2-II1) had the onset at age 8 with clinical symptoms of proximal muscle atrophy, beginning in the lower extremities, and weakness of the muscles in the shoulder and arms. Her CK level was measured at 16,408 U/L. However, her younger sister and brother are both healthy at 24 and 21 years old, respectively. Family 3 (F3) has two children (a girl and a boy) with the disease. The daughter (patient F3-II1) had the onset at the age of 13 with clinical symptoms of muscle weakness in the lower limbs. She lost the ability to walk at the age of 33. The brother (patient F3-II2) had the onset at the age of 11 with clinical symptoms: weakness of the muscles in the lower limbs and both arms. CK level measured at 5,000 U/L. Family 4 (F4) has one girl and one boy. Patient F4-II1 is a female, onset at the age of 10 years with clinical symptoms of weakness of the muscles of the lower extremities, buttocks, thighs, and difficulty standing up and sitting down, and a CK level of 1,257 U/L. She had surgery for lengthening the Achilles tendon at the age of 11. And at 18, she had proximal muscle weakness and lost the ability to walk. Her younger brother is 16 years old and still healthy. Muscle biopsies were analyzed in patients F1-II1, F1-II2 and F2-II1, F2-II3 including hematoxylin and eosin (H&E) stains (Figure 1).

**TABLE 1:** Clinical characteristics of enrolled patients in this study.

Patient	Age of onset (now)	Gender	CK level (U/L)	Clinical characteristics
F1-II1	7 (14)	Female	1,900	Weakness of the muscles in the shoulder and arms. Difficulties climbing stairs, standing up and sitting down. 8 years old: Lumbar lordosis appears
F1-II2	8 (9)	Male	16408.3	Proximal muscle atrophy, beginning in the lower extremities, and weakness of the muscles in the shoulder and arms. Difficulties climbing stairs, standing up and sitting down but not show lumbar lordosis
F2-II1	8 (29)	Female	16,408	Proximal muscle atrophy, beginning in the lower extremities, weakness of the muscles in the shoulder and arms
F3-II1	13 (33)	Female	1,607	Weakness of the muscles in the lower limbs. 33 years old: Lost the ability to walk
F3-II2	11 (31)	Male	5,000	Weakness of the muscles in the lower limbs. One year later, weakness of the muscles in both arms. Lost the ability to walk
F4-II1	10 (22)	Female	1,257	Weakness of the muscles of the lower extremities, buttocks, and thighs. Difficulties climbing stairs, standing up and sitting down. 11 years old: Achilles tendon lengthening surgery. 18 years old: Lost the ability to walk

### Genetic analysis

Genomic DNA (including the patients and their family members) was extracted using a Qiagen DNA blood mini kit (QIAGEN, Hilden, Germany) following manufacturer guidelines. Targeted sequencing was performed in patients using a gene panel for LGMD (Supplementary Table S2). The process was done on the Illumina sequencing machine (Illumina, CA, United States) and the data were analyzed based on the reference genome (GRCh38). Variants in LGMD-associated genes were screened to identify potentially pathogenic variants based on a minor allele frequency <0.01. CNV analysis was performed at Invitae Company using an in-house pipeline (Invitae, CA, United States).

Sanger sequencing was performed directly from PCR products using the primer pairs (designed with Primer3Plus software) on ABI PRISM 3500 Genetic Analyzer (Thermo Fisher Scientific Inc., United States) to confirm the detected variants. Sequencing data were analyzed using BioEdit 7.2.5 software based on ENSG00000108823 and ENSG00000092529 reference sequences for the *SGCA* and *CAPN3* genes in the ENSEMBL database, respectively. The novel variants were checked from dbSNP (https://www.ncbi.nlm.nih.gov/snp/), the 1,000 Genome Project (https://www.internationalgenome.org/1000-genomes-browsers/index.html), Exome Sequencing Project (https://evs.gs.washington.edu/EVS/), ExAC databases (https://gnomad.broadinstitute.org/), and the in-house database (*n* = 200). The influence of any novel nucleotide changes was evaluated with the *in silico* analysis tools: Fathmm (http://fathmm.biocompute.org.uk/inherited.html), M-CAP (Jagadeesh et al., 2016), Mutation Taster (https://www.mutationtaster.org/), PolyPhen 2 (http://genetics.bwh.harvard.edu/pph2/), PROVEAN (https://www.jcvi.org/research/provean), REVEL (Ioannidis et al., 2016), SIFT (https://sift.bii.a-star.edu.sg/). For splice mutation, the software prediction was used including MaxEntScan (http://hollywood.mit.edu/burgelab/maxent/Xmaxentscan_scoreseq.html), Spliceailookup (https://spliceailookup.broadinstitute.org/), Human Splicing Finder (https://bio.tools/human_splicing_finder), NetGene2 v. 2.42 (https://services.healthtech.dtu.dk/service.php?NetGene2-2.42), and Fruitfly (https://www.fruitfly.org/cgi-bin/seq_tools/splice.pl). ACMG guidelines (The American College of Medical Genetics and Genomics) (Richards et al., 2015) were used to interpret the pathogenicity of detected mutations.

## Results

In this study, six patients with LGMD from four unrelated Vietnamese families were collected for genetic analysis. The patients had the age of onset ranging from 7 to 13 years old, with similar specific clinical manifestations such as weakness in the shoulder, arms, and lower limb muscles; difficulty standing up and sitting down; and loss of the ability to walk within 18–33 years. In these patients, serum creatine kinase (CK) levels measured range from 1,257 U/L to 16,408 U/L, while the normal range was from 22 to 198 U/L (Table 1). Skeletal muscle biopsy from the patients (Figure 1) shows myopathic features including an increase of connective and adipose tissue (A: patient F1-II1), fiber size variation, and endomysial fibrofatty degeneration (B: patient F1-II2, D: patient F2-II3), and internalized nuclei (C: patient F2-II1). We conducted Targeted sequencing on the patient samples and identified five mutations in the genes: *SGCA* (NM_000023.4) (c.229C>T, p.Arg77Cys; exon one to three deletion (NC_000017.11(NM_000023.4):c.(?_-36)_(312 + 1_313-1)del); c.983 + 5G>C; and c.257_258insTGGCT, p.Phe88Leufs*125) and *CAPN3* (NM_000070.3) (c.946-4_946-1delACAG) (Table 2). The c.229C>T (p.Arg77Cys, rs28933693) mutation in the *SGCA* gene has been identified as a pathogenic mutation on the ClinVar database (under accession number VCV000009437.63). The c.946-4_946-1delACAG (rs766156798) mutation in the *CAPN3* gene has been also identified as a pathogenic mutation on the ClinVar database (under accession number VCV000497002.9). Two novel variants c.983+5G>C (Chr17-50170,671.G>C) and c.257_258insTGGCT (p.Phe88Leufs*125) in the *SGCA* gene were found in the patients. The c.983+5G>C variant in the *SGCA* gene is evaluated as a pathogenic mutation by the mutation taster software and as a Variant of Uncertain Significance (VUS) by the criteria in the evaluation table of The American College of Medical Genetics and Genomics (ACMG) (Table 3). The variant is also assessed as a damage mutation by MaxEntScan software with a score of −1.64 for mutant and a score of 8.49 for wildtype (Table 4). The c.257_258insTGGCT (p.Phe88Leufs*125) variant in the *SGCA* gene is evaluated as a pathogenic mutation by the mutation taster software and as a likely pathogenic mutation by the criteria in the evaluation table of ACMG (Table 3). The genetic analysis results showed that the patients in families one to three belong to the LGMDR3/2D subtype and patient in family 4 belong to the LGMDR1/2A subtype (Table 2).

**TABLE 2:** Mutations in alphasarcoglycan and calpain 3 identified in patients.

Patient	Gene	Nucleotide change (Amino acid change)	References (dbSNP)	Zygosity	Related diseases
F1-II1	*SGCA* (NM_000023.4)	c.229C>T (p.Arg77Cys)	rs28933693	Het	Type R3/2D
			Pathogenic		
		Deletion exon 1–3 (NC_000017.11(NM_000023.4):c.(?_-36)_(312 + 1_313-1)del)	Pathogenic	Het	
F1-II2	*SGCA* (NM_000023.4)	c.229C>T (p.Arg77Cys)	rs28933693	Het	Type R3/2D
			Pathogenic		
		Deletion exon 1-3 (NC_000017.11(NM_000023.4):c.(?_-36)_(312 + 1_313-1)del)	Pathogenic	Het	
F2-II1	*SGCA* (NM_000023.4)	c.983 + 5G>C	In this study	Hom	Type R3/2D
F3-II1	*SGCA* (NM_000023.4)	c.257_258insTGGCT (p.Phe88Leufs*125)	In this study	Hom	Type R3/2D
F3-II2	*SGCA* (NM_000023.4)	c.257_258insTGGCT (p.Phe88Leufs*125)	In this study	Hom	Type R3/2D
F4-II1	*CAPN3* (NM_000070.3)	c.946-4_946-1delACAG	rs766156798	Hom	Type R1/2A
			Pathogenic		

**TABLE 3:** Classification and evaluation of the mutations by *in silico* prediction software.

Family	Gene	Mutation	ACMG classification	ClinVar	Fathmm	Mutation taster	PROVEAN	SIFT	Pol-2	M-CAP	REVEL
F1	*SGCA*	c.229C>T (p.Arg77Cys)	P (PVS1+PM1+PM2)	P	P	P	P	P	P	P	P
		Deletion exon 1-3 (NC_000017.11(NM_000023.4):c.(?_-36)_(312 + 1_313-1)del)	P (PVS1+PM1+PM2)	NA	NA	NA	NA	NA	NA	NA	NA
F2	*SGCA*	c.983 + 5G>C	VUS (PM2+PP3+PP4)	NA	NA	P	NA	NA	NA	NA	NA
F3	*SGCA*	c.257_258insTGGCT (p.Phe88Leufs*125)	LP (PVS1+PM2+PP4)	NA	NA	P	NA	NA	NA	NA	NA
F4	*CAPN3*	c.946-4_946-1delACAG	P (PVS1+PM2+PP4)	P	NA	P	NA	NA	NA	NA	NA

ACMG, the american college of medical genetics and genomics; PROVEAN, protein variation effect analyzer; SIFT, sorting intolerant from tolerant; Pol-2, PolyPhen2; M-CAP, mendelian clinically applicable pathogenicity; REVEL, The rare exome variants ensemble learner; P, pathogenic; LP, likely pathogenic; VUS, variant of uncertain significance; NA, Not Available; PVS1, very strong; PS1-4, strong; PM1-6, moderate; PP1-5, supporting.

**TABLE 4:** Predictions from *in silico* software for the splice mutation c.983+5G>C.

*In silico* software	Wild type	Mutation	% Variation	Prediction
MaxEntScan	8.49	−1.64		Damage mutation
Spliceailookup	0.99	0.06		Donor loss
Human splicing finder	80.98	70.96	−12.36	Broken donor site
NetGene2	0.94	0.00		Donor loss
Fruitfly	0.93	0.00		Donor loss

## Discussion

In this study, six patients were collected from four unrelated Vietnamese families who were diagnosed with LGMD at the age of onset ranging from 7 to 13 years old. In all our patients, the initial symptoms were the weakness of the proximal muscles, followed by weakness of the muscles in the shoulder and arms. The patients have trouble climbing stairs, getting up, and sitting down from a squat. All patients had an early onset of the disease and loss of the ability to walk within 18–33 years. Previous studies have shown that patients with early-onset often progress rapidly muscle weakness leading to loss of independent mobility within 10 years. The review article by Vainzof et al. (2021) indicated that sarcoglycanopathies patients tend to present a severe clinical Duchenne muscular dystrophy-like, with early onset in childhood, and must be in a wheelchair before the age of sixteen; while, milder have also found in LGMDR5/2C, LGMDR3/2D, and LGMDR4/2E patients. Early-onset patients also had higher cardiovascular and respiratory complications than late-onset patients and had a life expectancy from adolescence to early adulthood (Georganopoulou et al., 2021). Cardiac complications may be more common in patients with delta and beta sarcoglycanopathies but have not been reported in patients with alpha and gamma sarcoglycanopathies (Angelini, 2020). The patients in our study also developed rapidly progressive muscle weakness and loss of mobility between the ages of 18 and 33. Cardiac and respiratory complications were not reported in our patients.

Gene analysis performed by Targeted sequencing revealed four mutations in the *SGCA* gene in five patients and one homozygous mutation in the *CAPN3* gene in one patient. Mutations occurring in any subunit of the sarcoglycan complex that can cause tissue damage have been identified as responsible for subtypes LGMDR3/2D - LGMDR6/2F (Al-Zaidy et al., 2015). Patients in the sarcoglycanopathy subtypes tend to present with symptoms in childhood and rapidly progress to loss of mobility during the early teen years. These patients have general symptoms such as abnormal gait, difficulty in running, climbing stairs, standing up, raising arms; big calves; and high CK levels. The *SGCA* gene was identified as the cause of LGMDR3/2D which is the common subtype among children. The first symptoms usually appear around 6 years of age (ages 1–30 years) in all subtypes except in LGMDR3/2D subtype at age 13 (Narayanaswami et al., 2014; Alonso-Pérez et al., 2020).

**FIGURE 2:**
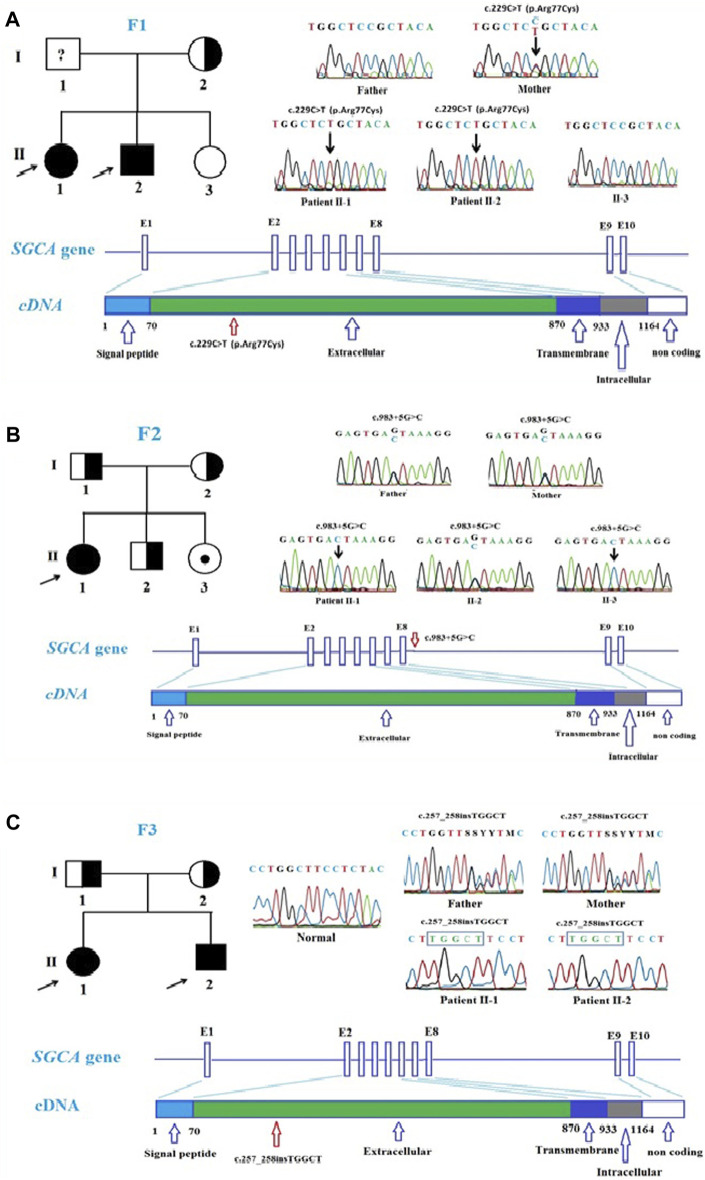
Mutations in *SGCA* gene in the patients. **(A)**. Pedigree of family 1 and Sanger sequencing results at the mutation c.229C>T, p.Arg77Cys in the patient and the patient’s family members. **(B)**. Pedigree of family 2 and Sanger sequencing results at the mutation c.983 + 5G>C in the patient and the patient’s family members. **(C)**. Pedigree of family 3 and Sanger sequencing results at the mutation c.257_258insTGGCT, p.Phe88Leufs*125 in the patient and the patient’s family members.

In family 1, by Targeted sequencing, the compound heterozygous of exon one to three deletion (NC_000017.11 (NM_000023.4):c.(?_-36)_(312 + 1_313-1)del) mutation and missense (c.229C>T, p.Arg77Cys) mutation were detected in the *SGCA* gene in two patients. Sanger sequencing results showed that two patients in family 1 (F1-II1 and F1-II2) inherited the (c.229C>T, p.Arg77Cys) mutation in the *SGCA* gene from their mother (Figure 2A). This mutation has been evaluated as a pathogenic mutation in the ClinVar database (Table 3). The c.229C>T (p.Arg77Cys) mutation is also reported to be one of the most common genetic variants of LGMDR3/2D, causing protein synthesis in the ER that prevents the formation of the sarcoglycan complex (Georganopoulou et al., 2021). However, in this study, we did not perform an MLPA assessment to confirm the exon one to three deletion (NC_000017.11(NM_000023.4):c.(?_-36)_(312 + 1_313-1)del) mutation on the members of family 1. We hypothesized that the exon one to three deletion (NC_000017.11(NM_000023.4):c.(?_-36)_(312 + 1_313-1)del) mutation in two patients was inherited from their father. This mutation is evaluated as a pathogenic mutation by the criteria in the evaluation table of ACMG (Table 3). Skeletal muscle biopsy from the patients (Figures 1A, B) showed that there have been changes in the patient’s muscle tissue structure includ*ing an* increase of connective and adipose tissue, fiber size variation, and endomysial fibrofatty degeneration.

In family 2, Sanger sequencing results showed that the patient carried the novel homozygous NC_000017.11(NM_000023.4):c.983 + 5G>C variant in the *SGCA* gene inherited from the parents who harbor a heterozygous mutation (Figure 2B). The special thing is that the patient’s sister (a 21-year-old girl) who also carried this homozygous variant, but has not yet shown any symptoms of the disease. This variant is evaluated by the mutation taster software as a pathogenic mutation (Table 3). The variant is determined as a damage mutation by MaxEntScan software with a score of −1.64 for the mutant and a score of 8.49 for the wildtype (Table 4). The evaluation results of the Human Splicing Finder software show that the variant leads to a broken donor site and affects the splicing process. Prediction by NetGene2 v.2.42 and Fruitfly software show that the variant leads to the loss of the donor site. Two software also make predictions for a new donor site at position 1,244 nucleotides away from the original site (Supplementary Table S3A). However, the variant is evaluated as a Variant of Uncertain Significance (VUS) by the criteria in the evaluation table of ACMG (Table 3). In addition, a study by Xie et al. (2019) on 218 LGMD patients showed that there was a statistically significant positive correlation between reduced α-SG level and disease severity in the patients with LGMDR3/2D and indicated that the higher the residual protein, the milder the disease. In a study by Xie et al. (2019), patient 12, harbored a canonical splicing c.956 + 2T>C mutation in the *SGCA* gene, and showed a slight reduction in α-SG level on immunohistochemical staining. Muscle biopsy from the patient F2-II1 (Figure 1C) showed that there have been changes in the muscle structure *including* fiber size variation, internal nuclei, and *an* increase of connective and adipose tissue. Interestingly although the patient’s sister (F2-II3) has not yet presented the disease, the changes in fiber size variation and endomysial fibrofatty degeneration were observed in the image of the muscle biopsy (Figure 1D). These results provide evidence that the patient’s sister (F2-II3) has a high risk to developing the disease.

In family 3, two patients carried the novel homozygous (c.257_258insTGGCT, p.Phe88Leufs*125) mutation in the *SGCA* gene. The homozygous genotype in the patient was inherited from their parents who carried the heterozygous mutation (Figure 2C). The frameshift mutation leads to producing a truncated protein with no function. The mutation is evaluated as a pathogenic mutation by the mutation taster software and as a likely pathogenic mutation by the criteria in the evaluation table of ACMG (Table 3). Although there is no direct correlation between genotype and phenotype, nonsense mutations are often thought to lead to severe phenotypes (Georganopoulou et al., 2021). This also explains the cause of the disease in the patients.

**FIGURE 3:**
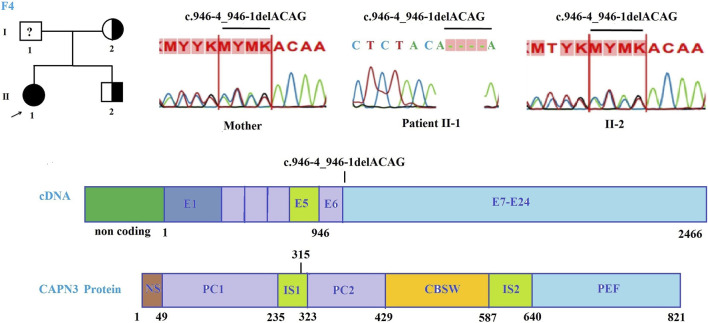
Mutations in the *CAPN3* gene in the patient of family 4. Pedigree of family 4 and Sanger sequencing results at the mutation c.946-4_946-1delACAG in the patient and the patient’s family members.

In family 4, Sanger sequencing results showed that the patient carried the homozygous c.946-4_946-1delACAG mutation in the *CAPN3* gene, and the patient’s mother carried the heterozygous for this mutation (Figure 3). However, a sample of the patient’s father could not be collected, so his genotype could not be determined. The mutation is evaluated as a pathogenic mutation in the ClinVar database and by the mutation taster software as well as by the criteria in the evaluation table of ACMG (Table 3). The mutation occurred at intron 7 and was predicted to make a new acceptor site at 175 nucleotides from the original site using NetGene2 v.2.42 and Fruitfly software (Supplementary Table S3B). The mutation may be responsible for the skipping of exon 7 of the *CAPN3* gene. Homozygous or compound heterozygous mutations in the *CAPN3* gene are known to be the cause of the LGMDR1/2A subtype, a rare form that usually appears in children and young adults, results in loss of ambulation within 20 years in most patients (Lasa-Elgarresta et al., 2019). Recently, heterozygous mutations in the *CAPN3* gene were identified as the cause of autosomal dominant limb-girdle muscular dystrophy-4 (LGMDD4), with a later onset and milder phenotype (Vissing et al., 2016; Martinez-Thompson et al., 2018a; b), although the pathogenicity of these cases remains elucidated (Sáenz and López de Munain, 2017). The results of the genetic analysis showed that the patient carried a homozygous mutation in the *CAPN3* gene, which again confirmed that the patient belonged to the LGMDR1/2A subtype. The patient F4-II1 has an age onset of 10 years with clinical symptoms of weakness of the muscles of the lower extremities, buttocks, and thighs. The patient had Achilles tendon lengthening surgery at 11 years old and lost the ability to walk at 18 years old. The patient’s clinical manifestations were completely consistent with the description in patients with calpainopathy including the first symptoms start in the lower and upper limbs, the disease progression is considered moderate in the LGMD subtypes, and the ability to walk is usually lost around age 35 or in the first 20 years of life (Cotta et al., 2014; Lasa-Elgarresta et al., 2019).

## Conclusion

In this study, Targeted sequencing was performed to identify mutations in six patients with LGMD from four unrelated Vietnamese families. Five mutations including four (c.229C>T, p.Arg77Cys; exon one to three deletion; c.983+5G>C; and c.257_258insTGGCT, p.Phe88Leufs*125) in the *SGCA* gene and one (c.946-4_946-1delACAG) in the *CAPN3* gene were detected in the patients. Two homozygous mutations (c.983+5G>C and c.257_258insTGGCT) in the *SGCA* gene were novel. Our results have contributed to the general understanding of the etiology of the disease for diagnosis purposes as well as to support genetic counseling and prenatal screening.

## Data Availability

The data presented in the study are deposited in the LOVD database (https://databases.lovd.nl/shared/variants/), under accession numbers: 0000434890 - 0000434894 and 0000438149.
