# Supplementary material for: Novel mutations in the SGCA gene in unrelated Vietnamese patients with limb-girdle muscular dystrophies disease
Source: Front Genet. 2023 Oct 13;14:1248338. doi: 10.3389/fgene.2023.1248338 (PMC10611451; doi:10.3389/fgene.2023.1248338)
Supplement: Supplementary file 1 [file Table1.DOCX]

**Table S1.** LGMD subtypes and related genes

| **LGMD subtypes**  **(OMIM)** | **Gene (OMIM)** | **Chr** | **Protein** |
| --- | --- | --- | --- |
| **Autosomal dominant** |  |  |  |
| D1 (1D) (603511) | *DNAJB6* (611332) | 7q36.3 | DNAJB6 |
| D2 (1F) (608423) | *TPNO3* (610032) | 7q32.1 | Transportin-3 |
| D3 (1G) (609115) | *HNRPDL* (607137) | 4p21.22 | Heterogeneous molecular ribonucleic D-like protein |
| D4 (1I) (618129) | *CAPN3* (114240) | 15q15.1 | Calpain-3 |
| D5 (1K) (158810) | *COL6A1/2/3* | 21q22.3 | Collagen VI subunits A1, A2, or A3 |
| **Autosomal recessive** |  |  |  |
| R1 (2A) (253600) | *CAPN3* (114240) | 15q15.1 | Calpain-3 |
| R2 (2B) (253601) | *DYSF* (603009) | 2p13.2 | Dysferlin |
| R3 (2D) (608099) | *SGCA* (600119) | 17q21.33 | a-sarcoglycan |
| R4 (2E) (604286) | *SGCB* (600900) | 4q12 | b-sarcoglycan |
| R5 (2C) (253700) | *SGCG* (608896) | 13q12.12 | g-sarcoglycan |
| R6 (2F) (601287) | *SGCD* (601411) | 5q33.3 | d-sarcoglycan |
| R7 (2G) (601954) | *TCAP* (604488) | 17q12 | Telethonin |
| R8 (2H) (254110) | *TRIM32* (602290) | 9q33.1 | Tripartite motif containing protein-32 |
| R9 (2I) (607155) | *FKRP* (606596) | 19q13.32 | Fukutin-related-protein |
| R10 (2J) (608807) | *TTN* (188840) | 2q31.2 | Titin |
| R11 (2K) (609308) | *POMT1* (607423) | 9q34.13 | Protein O-mannosyl transferase-1 |
| R12 (2L) (611307) | *ANO5* (608662) | 11p14.3 | Anoctamin-5 |
| R13 (2M) (611588) | *FKTN* (607440) | 9q31.2 | Fukutin |
| R14 (2N) (613158) | *POMT2* (607439) | 14q24.3 | Protein O-mannosyl transferase-2 |
| R15 (2O) (613157) | *POMGnT1* (606822) | 1p34.1 | Protein O-mannose N-acetyl-glucosaminyl Transferase-1 |
| R16 (2P) (613818) | *DAG1* (128239) | 3p21 | Dystroglycan |
| R17 (2Q) (613723) | *PLEC1* (601282) | 8q24.3 | Plectin |
| R18 (2S) (615356) | *TRAPPC11* (614138) | 4q35.1 | Transport-protein-particle-complex-11 |
| R19 (2T) (615352) | *GMPPB* (615320) | 3p21.31 | GDP-mannose-pyrophosphorylase B |
| R20 (2U) (616052) | *CRPPA/ISPD* | 7p21 | CDP-L-ribitol pyrophosphorylase A |
| R21 (2Z) (617232) | *POGLUT1* (615618) | 3q13.33 | Protein-O-glucosyltransferase-1 |
| R22 (254090) | *COL6A1/2/3* | 21q22.3 | Collagen VI subunits A1, A2, or A3 |
| R23 (618138) | *LAMA2* (156225) | 6q22 | Laminin subunit alpha 2 |
| R24 (618135) | *POMGNT2*  (614828) | 3p22 | Protein O-linked mannose N-acetylglucosaminyltransferase 2  (beta 1,4-) |
| R25 (2X) (616812) | *BVES* (604577) | 6q21 | Blood vessel epicardial substance |
| R26 (618848) | *POPDC3* (605824) | 6q21 | Popeye domain containing protein 3 |
| R27 (619566) | *JAG2* (602570) | 14q32 | Jagged 2 |
| R (number pending)  (617258) | *PYROXD1* (617220) | 12p12.1 | Nuclear-cytoplasmic pyridine nucleotide-disulphide reductase |
