# Supplementary material for: Novel mutations in the SGCA gene in unrelated Vietnamese patients with limb-girdle muscular dystrophies disease
Source: Front Genet. 2023 Oct 13;14:1248338. doi: 10.3389/fgene.2023.1248338 (PMC10611451; doi:10.3389/fgene.2023.1248338)
Supplement: Supplementary file 2 [file Table2.DOCX]

**Table S2**. List of genes in the gene panel

| **No** | **Diseases’ name** | **Gene** |
| --- | --- | --- |
| **Hereditary Muscular Disease** | | |
| 1 | Nemaline Myopathy 1 | *TPM3* |
| 2 | Nemaline Myopathy 2 | *NEB* |
| 3 | Nemaline Myopathy 3 | *ACTA1* |
| 4 | Nemaline Myopathy 4 | *TPM2* |
| 5 | Nemaline Myopathy 5 | *TNNT1* |
| 6 | Nemaline Myopathy 6 | *KBTBD13* |
| 7 | Nemaline Myopathy 7 | *CFL2* |
| 8 | Salih Myopathy | *TTN* |
| 9 | Deafness-Dystonia-Optic Neuronopathy Syndrome | *TIMM8A* |
| 10 | Myoclonus-Dystonia | *SGCE* |
| 11 | Myopathy with Deficiency of ISCU | *ISCU* |
| 12 | X-Linked Centronuclear Myopathy | *MTM1* |
| 13 | Hereditary Inclusion Body Myopathy 2 | *GNE* |
| 14 | Inclusion Body Myopathy with Paget Disease of Bone&Frontotemporal Dementia | *VCP* |
| 15 | Myotonia Congenita | *CLCN1* |
| 16 | Paramyotonia Congenita | *SCN4A* |
| 17 | Potassium-aggravated myotonia | *SCN4A* |
| 18 | Dopa-Responsive Dystonia | *GCH1、SPR、TH* |
| 19 | Dystonia&Parkinsonism, Hypermanganesemia, Polycythemia, and Chronic Liver Disease | *SLC30A10* |
| 20 | Rapid-Onset Dystonia-Parkinsonism | *ATP1A3* |
| 21 | Early-Onset Primary Dystonia | *TOR1A* |
| 22 | Central Core Disease | *RYR1* |
| 23 | Multiminicore Disease | *RYR1* |
| 24 | Miyoshi Distal Myopathy (Miyoshi Myopathy) | *DYSF* |
| 25 | Myosin Storage Myopathy | *MYH7* |
| 26 | Mitochondrial Myopathy And Sideroblastic Anemia | *PUS1* |
| 27 | Cap Myopathy 1 | *ACTA1* |
| 28 | Cap Myopathy 2 | *TPM2* |
| 29 | Cap Myopathy 3 | *TPM3* |
| 30 | Collagen VI-deficient Congenital Muscular Dystrophy | *COL6A1、COL6A2、COL6A3* |
| 31 | Brody Myopathy | *ATP2A1* |
| 32 | Laing Distal Myopathy | *MYH7* |
| 33 | Distal Myopathy 2 | *MATR3* |
| 34 | Distal Myopathy 4 | *FLNC* |
| 35 | Hyperkalemic Periodic Paralysis | *SCN4A* |
| 36 | Hypokalemic Periodic Paralysis | *CACNA1S、SCN4A* |
| 37 | ACTA1-Related Congenital Fiber-Type Disproportion | *ACTA1* |
| 38 | SEPN1-Related Congenital Fiber-Type Disproportion | *SEPN1* |
| 39 | TPM3-Related Congenital Fiber-Type Disproportion | *TPM3* |
| 40 | TPM2-Related Congenital Fiber-Type Disproportion | *TPM2* |
| 41 | MYH7-Related Congenital Fiber-Type Disproportion | *MYH7* |
| 42 | RYR1-Related Congenital Fiber-Type Disproportion | *RYR1* |
| 43 | LAMA2-Related Congenital Muscular Dystrophy | *LAMA2* |
| 44 | Collagen VI-deficient Congenital Muscular Dystrophy | *COL6A1、COL6A2、COL6A3* |
| 45 | SEPN1-related Congenital Muscular Dystrophy | *SEPN1* |
| 46 | SYNE1-related Congenital Muscular Dystrophy | *SYNE1* |
| 47 | Duchenne Muscular Dystrophy | *DMD* |
| 48 | Becker Muscular Dystrophy | *DMD* |
| 49 | Muscular dystrophy-dystroglycanopathy type B1 | *POMT1* |
| 50 | Muscular dystrophy-dystroglycanopathy type B2 | *POMT2* |
| 51 | Muscular dystrophy-dystroglycanopathy type B4 | *FKTN* |
| 52 | Muscular dystrophy-dystroglycanopathy type B5 | *FKRP* |
| 53 | Muscular dystrophy-dystroglycanopathy type B6 | *LARGE1* |
| 54 | Muscular dystrophy-dystroglycanopathy type B3 | *POMGNT1* |
| 55 | Muscular dystrophy-dystroglycanopathy type A7 | *ISPD* |
| 56 | Muscular dystrophy-dystroglycanopathy type A5 | *FKRP* |
| 57 | Fukuyama congenital muscular dystrophy | *FKTN* |
| 58 | Limb-Girdle Muscular Dystrophy type 2C | *SGCG* |
| 59 | Limb-Girdle Muscular Dystrophy type 2D | *SGCA* |
| 60 | Limb-Girdle Muscular Dystrophy type 2E | *SGCB* |
| 61 | Limb-Girdle Muscular Dystrophy type 2F | *SGCD* |
| 62 | Limb-Girdle Muscular Dystrophy type 2A | *CAPN3* |
| 63 | Limb-Girdle Muscular Dystrophy type 2B | *DYSF* |
| 64 | Limb-Girdle Muscular Dystrophy type 2G | *TCAP* |
| 65 | Limb-Girdle Muscular Dystrophy type 2H | *TRIM32* |
| 66 | Limb-Girdle Muscular Dystrophy type 2I | *FKRP* |
| 67 | Limb-Girdle Muscular Dystrophy type 2K | *POMT1* |
| 68 | Limb-Girdle Muscular Dystrophy type 2J | *TTN* |
| 69 | Limb-Girdle Muscular Dystrophy type 2L | *ANO5* |
| 70 | Limb-Girdle Muscular Dystrophy type 2M | *FKTN* |
| 71 | Limb-Girdle Muscular Dystrophy type 2N | *POMT2* |
| 72 | Limb-Girdle Muscular Dystrophy type 2O | *POMGNT1* |
| 73 | Limb-Girdle Muscular Dystrophy type 2Q | *PLEC* |
| 74 | Limb-Girdle Muscular Dystrophy type 2R | *DES* |
| 75 | Limb-Girdle Muscular Dystrophy type 2S | *TRAPPC11* |
| 76 | Limb-Girdle Muscular Dystrophy type 1A | *MYOT* |
| 77 | Limb-Girdle Muscular Dystrophy type 1B | *LMNA* |
| 78 | Limb-Girdle Muscular Dystrophy type 1C | *CAV3* |
| 79 | Limb-Girdle Muscular Dystrophy type 1D | *DES* |
| 80 | Limb-Girdle Muscular Dystrophy type 1E | *DNAJB6* |
| 81 | X-linked Emery-Dreifuss Muscular Dystrophy 1 | *EMD* |
| 82 | X-linked Emery-Dreifuss Muscular Dystrophy 6 | *FHL1* |
| 83 | Emery-Dreifuss muscular dystrophy 2 | *LMNA* |
| 84 | Emery-Dreifuss muscular dystrophy 3 | *LMNA* |
| 85 | Emery-Dreifuss muscular dystrophy 4 | *SYNE1* |
| 86 | Emery-Dreifuss muscular dystrophy 5 | *SYNE2* |
| 87 | Emery-Dreifuss muscular dystrophy 7 | *TMEM43* |
| 88 | Congenital myasthenic syndrome with tubular aggregates 2 | *DPAGT1* |
| 89 | AGRN-Related Congenital Myasthenic Syndrome | *AGRN* |
| 90 | CHAT-Related Congenital Myasthenic Syndrome | *CHAT* |
| 91 | CHRNA1-Related Congenital Myasthenic Syndrome | *CHRNA1* |
| 92 | CHRNB1-Related Congenital Myasthenic Syndrome | *CHRNB1* |
| 93 | CHRND-Related Congenital Myasthenic Syndrome | *CHRND* |
| 94 | CHRNE-Related Congenital Myasthenic Syndrome | *CHRNE* |
| 95 | COLQ-Related Congenital Myasthenic Syndrome | *COLQ* |
| 96 | DOK7-Related Congenital Myasthenic Syndrome | *DOK7* |
| 97 | GFPT1-Related Congenital Myasthenic Syndrome | *GFPT1* |
| 98 | MUSK-Related Congenital Myasthenic Syndrome | *MUSK* |
| 99 | RAPSN-Related Congenital Myasthenic Syndrome | *RAPSN* |
| 100 | SCN4A-Related Congenital Myasthenic Syndrome | *SCN4A* |
| 101 | Hyperkalemic Periodic Paralysis | *SCN4A* |
| 102 | Hypokalemic Periodic Paralysis | *CACNA1S、SCN4A* |
| 103 | Myofibrillar Myopathy 1 | *DES* |
| 104 | CRYAB-related Myofibrillar Myopathy | *CRYAB* |
| 105 | Myofibrillar Myopathy 3 | *MYOT* |
| 106 | Myofibrillar Myopathy 4 | *LDB3* |
| 107 | Myofibrillar Myopathy 5 | *FLNC* |
| 108 | Myofibrillar Myopathy 6 | *BAG3* |
| 109 | FHL1-Related Myofibrillar Myopathy | *FHL1* |
| 110 | DNAJB6-Related Myofibrillar Myopathy | *DNAJB6* |
| 111 | Autosomal Recessive Progressive external ophthalmoplegia | *POLG* |
| 112 | Adenosine Monophosphate Deaminase Deficiency | *AMPD1* |
| 113 | Danon disease | *LAMP2* |
| 114 | Pseudocholinesterase deficiency | *BCHE* |
| 115 | X-Linked Dystonia-Parkinsonism | *TAF1* |
| **Hereditary Ataxias due to non-dynamic mutation** | | |
| 116 | Episodic Ataxia Type 1 | *KCNA1* |
| 117 | Episodic Ataxia Type 5 | *CACNB4* |
| 118 | Episodic Ataxia Type 6 | *SLC1A3* |
| 119 | Spastic ataxia 1 | *VAMP1* |
| 120 | Ataxia-telangiectasia | *ATM* |
| 121 | Ataxia with vitamin E deficiency | *TTPA* |
| 122 | Ataxia with oculomotor apraxia type 1 | *APTX* |
| 123 | Ataxia with oculomotor apraxia type 2 | *SETX* |
| 124 | Infantile-Onset Spinocerebellar Ataxia | *C10orf2* |
| 125 | Marinesco-Sjögren syndrome | *SIL1* |
| 126 | Neuroferritinopathy | *FTL* |
| 127 | Myoclonic Epilepsy Myopathy Sensory Ataxia | *POLG* |
| 128 | Autosomal Recessive Spastic Ataxia of Charlevoix-Saguenay | *SACS* |
| 129 | Autosomal Recessive Spinocerebellar Ataxia with Axonal Neuropathy | *TDP1* |
| 130 | Cerebrotendinous xanthomatosis | *CYP27A1* |
| 131 | X-linked sideroblastic anemia and ataxia | *ABCB7* |
| 132 | PRICKLE1-Related Progressive Myoclonus Epilepsy with Ataxia | *PRICKLE1* |
| **Familial Amyotrophic Lateral Sclerosis** | | |
| 133 | Familial Amyotrophic Lateral Sclerosis 1 | *SOD1* |
| 134 | Familial Amyotrophic Lateral Sclerosis 2 | *ALS2* |
| 135 | Familial Amyotrophic Lateral Sclerosis 4 | *SETX* |
| 136 | Familial Amyotrophic Lateral Sclerosis 6 | *FUS* |
| 137 | Familial Amyotrophic Lateral Sclerosis 8 | *VAPB* |
| 138 | Familial Amyotrophic Lateral Sclerosis 9 | *ANG* |
| 139 | Familial Amyotrophic Lateral Sclerosis 10 | *TARDBP* |
| 140 | Familial Amyotrophic Lateral Sclerosis 11 | *FIG4* |
| 141 | Familial Amyotrophic Lateral Sclerosis 12 | *OPTN* |
| 142 | Familial Amyotrophic Lateral Sclerosis 14 | *VCP* |
| 143 | Familial Amyotrophic Lateral Sclerosis 15 | *UBQLN2* |
| 144 | Familial Amyotrophic Lateral Sclerosis 16 | *SIGMAR1* |
| 145 | Familial Amyotrophic Lateral Sclerosis 17 | *CHMP2B* |
| 146 | Familial Amyotrophic Lateral Sclerosis 18 | *PFN1* |
| 147 | Familial Amyotrophic Lateral Sclerosis 20 | *HNRNPA1* |
| 148 | Familial Amyotrophic Lateral Sclerosis 21 | *MATR3* |
| 149 | Troyer syndrome | *SPG20* |
