# Supplementary material for: Novel mutations in the SGCA gene in unrelated Vietnamese patients with limb-girdle muscular dystrophies disease
Source: Front Genet. 2023 Oct 13;14:1248338. doi: 10.3389/fgene.2023.1248338 (PMC10611451; doi:10.3389/fgene.2023.1248338)
Supplement: Supplementary file 3 [file Table3.DOCX]

**Table S3.** Results from *in silico* analysis of the splice mutation.

| **A. c.983+5G>C in the *SGCA* gene** | | | | | | | |
| --- | --- | --- | --- | --- | --- | --- | --- |
| **NetGene2 v. 2.42** | | | | | | | |
| **Wild type** | | | | **Mutation** | | | |
| Pos  5'-3' | Confi-  dence | 5' exon intron | | Pos  5'-3' | Confi-  dence | 5' exon intron | |
| 531 | **0.89** | CTACCTCCGA^GTGAGTAAAG | |  |  |  | |
| 1775 | **0.91** | TTTGGCACAG^GTGCGTTGCC | | 1775 | **0.91** | TTTGGCACAG^GTGCGTTGCC | |
| 1901 | 0.47 | TCTCCCTGGG^GTAGGTGAAG | | 1901 | 0.47 | TCTCCCTGGG^GTAGGTGAAG | |
| 2297 | 0.00 | GGCCTGCACG^GTGCGTGCAC | | 2297 | 0.00 | GGCCTGCACG^GTGCGTGCAC | |
| **Fruitfly** | | | | | | | |
| **Wild type** | | | | **Mutation** | | | |
| Start - End | | Score | 5' exon intron | Start - End | | Score | 5' exon intron |
| 108 - 122 | | **0.93** | cctccga**gt**gagtaa |  | |  |  |
| 320 - 334 | | 0.60 | caacaca**gt**gagacc | 320 - 334 | | 0.60 | caacaca**gt**gagacc |
| 1352 - 1366 | | 0.89 | ggcacag**gt**gcgttg | 1352 - 1366 | | 0.89 | ggcacag**gt**gcgttg |
| 1478 - 1492 | | 0.71 | ccctggg**gt**aggtga | 1478 - 1492 | | 0.71 | ccctggg**gt**aggtga |
| 1558 - 1572 | | **0.96** | ggctgag**gt**gtgtcc | 1558 - 1572 | | **0.96** | ggctgag**gt**gtgtcc |
| 1874 - 1888 | | 0.97 | ctgcacg**gt**gcgtgc | 1874 - 1888 | | 0.97 | ctgcacg**gt**gcgtgc |
| 2840 - 2854 | | 0.44 | aggccac**gt**gtgtgt | 2840 - 2854 | | 0.44 | aggccac**gt**gtgtgt |
| 3579 - 3593 | | 0.84 | caacata**gt**aagaca | 3579 - 3593 | | 0.84 | caacata**gt**aagaca |
| 3692 - 3706 | | 0.47 | ccaggag**gt**agaggc | 3692 - 3706 | | 0.47 | ccaggag**gt**agaggc |
| 3745 - 3759 | | 0.84 | cgacaaa**gt**gagacc | 3745 - 3759 | | 0.84 | cgacaaa**gt**gagacc |
| 3945 - 3959 | | 0.94 | ctaggag**gt**aggttt | 3945 - 3959 | | 0.94 | ctaggag**gt**aggttt |
| 4066 - 4080 | | 0.85 | attacag**gt**gtgagc | 4066 - 4080 | | 0.85 | attacag**gt**gtgagc |
| 4093 - 4107 | | 0.69 | cctggtg**gt**aggtgt | 4093 - 4107 | | 0.69 | cctggtg**gt**aggtgt |
| 4329 - 4343 | | 0.46 | ctcccag**gt**tcaagt | 4329 - 4343 | | 0.46 | ctcccag**gt**tcaagt |

<https://services.healthtech.dtu.dk/service.php?NetGene2-2.42>

********************** NetGene2 v. 2.42 ************************

The sequence: **Normal** has the following composition:

Length: 2340 nucleotides.

19.1% A, 28.5% C, 29.5% G, 22.9% T, 0.0% X, 58.0% G+C

Donor splice sites, direct strand ---------------------------------

pos 5'->3' phase strand confidence 5' exon intron 3'

216 2 + 0.94 GGGAGGGAAG^GTGAATGTGG H

531 0 + 0.89 CTACCTCCGA^GTGAGTAAAG

1775 0 + 0.91 TTTGGCACAG^GTGCGTTGCC H

1901 1 + 0.47 TCTCCCTGGG^GTAGGTGAAG

2297 1 + 0.00 GGCCTGCACG^GTGCGTGCAC

********************** NetGene2 v. 2.42 ************************

The sequence: **Mutant** has the following composition:

Length: 2340 nucleotides.

19.1% A, 28.5% C, 29.5% G, 22.9% T, 0.0% X, 58.0% G+C

Donor splice sites, direct strand ---------------------------------

pos 5'->3' phase strand confidence 5' exon intron 3'

216 2 + 0.94 GGGAGGGAAG^GTGAATGTGG H

1775 0 + 0.91 TTTGGCACAG^GTGCGTTGCC H

1901 1 + 0.47 TCTCCCTGGG^GTAGGTGAAG

2297 1 + 0.00 GGCCTGCACG^GTGCGTGCAC

<https://www.fruitfly.org/cgi-bin/seq_tools/splice.pl>

### Donor site predictions for wild :

**Start End Score Exon Intron**
 108 122 0.93 cctccgagtgagtaa
 320 334 0.60 caacacagtgagacc
 1352 1366 0.89 ggcacaggtgcgttg
 1478 1492 0.71 ccctggggtaggtga
 1558 1572 0.96 ggctgaggtgtgtcc
 1874 1888 0.97 ctgcacggtgcgtgc
 2840 2854 0.44 aggccacgtgtgtgt
 3579 3593 0.84 caacatagtaagaca
 3692 3706 0.47 ccaggaggtagaggc
 3745 3759 0.84 cgacaaagtgagacc
 3945 3959 0.94 ctaggaggtaggttt
 4066 4080 0.85 attacaggtgtgagc
 4093 4107 0.69 cctggtggtaggtgt
 4329 4343 0.46 ctcccaggttcaagt

### Donor site predictions for mutant :

**Start End Score Exon Intron**
 320 334 0.60 caacacagtgagacc
 1352 1366 0.89 ggcacaggtgcgttg
 1478 1492 0.71 ccctggggtaggtga
 1558 1572 0.96 ggctgaggtgtgtcc
 1874 1888 0.97 ctgcacggtgcgtgc
 2840 2854 0.44 aggccacgtgtgtgt
 3579 3593 0.84 caacatagtaagaca
 3692 3706 0.47 ccaggaggtagaggc
 3745 3759 0.84 cgacaaagtgagacc
 3945 3959 0.94 ctaggaggtaggttt
 4066 4080 0.85 attacaggtgtgagc
 4093 4107 0.69 cctggtggtaggtgt
 4329 4343 0.46 ctcccaggttcaagt

| **B. c.946-4_946-1delACAG in the *CAPN3* gene** | | | | | | | | | |
| --- | --- | --- | --- | --- | --- | --- | --- | --- | --- |
| **NetGene2 v. 2.4** | | | | | | | | | |
| **Wild type** | | | | | **Mutation** | | | | |
| Pos  5'-3' | Confi-  dence | | 5' exon intron | | Pos  5'-3' | Confi-  dence | 5' exon intron | | |
| 509 | 0.16 | | TATGTTCCAG^GCACTCCTCT | | 509 | 0.16 | TATGTTCCAG^GCACTCCTCT | | |
| 729 | 0.25 | | GTTCTTCCAG^GATTTTTCTA | | 729 | 0.25 | GTTCTTCCAG^GATTTTTCTA | | |
| 1473 | 0.07 | | GATTAATGAG^TCTCCCGCCC | | 1473 | 0.07 | GATTAATGAG^TCTCCCGCCC | | |
| 1491 | 0.67 | | CCCCTCCCAG^TCTATTCAGC | | 1491 | 0.67 | CCCCTCCCAG^TCTATTCAGC | | |
| 1500 | 0.31 | | GTCTATTCAG^CATGATCTGG | | 1500 | 0.31 | GTCTATTCAG^CATGATCTGG | | |
| 2296 | 0.77 | | TCTCTTCCAG^GAATAGTCAA | | 2296 | 0.77 | TCTCTTCCAG^GAATAGTCAA | | |
| 2381 | 0.29 | | TCCTTTGAAG^GATACTAAGG | | 2381 | 0.29 | TCCTTTGAAG^GATACTAAGG | | |
| 2593 | 0.43 | | GTCTTCACAG^AGCCCGGAAA | | 2593 | 0.43 | GTCTTCACAG^AGCCCGGAAA | | |
| 2686 | 0.39 | | TGCTCTACAG^ACAATCATTC | | 2634 | 0.07 | CTCCAAGCAG^CAGAACTTCT | | |
| **Fruitfly** | | | | | | | | | |
| **Wild type** | | | | | **Mutation** | | | | |
| Start - End | | Score | | 5' exon intron | Start - End | | | Score | 5' exon intron |
| 489 - 529 | | 0.79 | | atgttcc**ag**gcactc | 489 - 529 | | | 0.79 | atgttcc**ag**gcactc |
| 566 - 606 | | 0.68 | | ctcttgt**ag**agttta | 566 - 606 | | | 0.68 | ctcttgt**ag**agttta |
| 709 - 749 | | 0.78 | | ttcttcc**ag**gatttt | 709 - 749 | | | 0.78 | ttcttcc**ag**gatttt |
| 783 - 823 | | 0.84 | | tctttgc**ag**cctgct | 783 - 823 | | | 0.84 | tctttgc**ag**cctgct |
| 797 - 837 | | 0.69 | | ttttttc**ag**ttagtt | 797 - 837 | | | 0.69 | ttttttc**ag**ttagtt |
| 863 - 903 | | 0.94 | | attccct**ag**gctgga | 863 - 903 | | | 0.94 | attccct**ag**gctgga |
| 937 - 977 | | 0.65 | | ctgcctc**ag**cctccc | 937 - 977 | | | 0.65 | ctgcctc**ag**cctccc |
| 1005 - 1045 | | 0.96 | | ttttagt**ag**agacgg | 1005 - 1045 | | | 0.96 | ttttagt**ag**agacgg |
| 1814 - 1854 | | 0.71 | | ctttctt**ag**aattct | 1814 - 1854 | | | 0.71 | ctttctt**ag**aattct |
| 1866 - 1906 | | 0.84 | | atgttcc**ag**gtgggt | 1866 - 1906 | | | 0.84 | atgttcc**ag**gtgggt |
| 2276 - 2316 | | 0.99 | | ctcttcc**ag**gaatag | 2276 - 2316 | | | 0.99 | ctcttcc**ag**gaatag |
| 2361 - 2401 | | 0.94 | | cctttga**ag**gatact | 2361 - 2401 | | | 0.94 | cctttga**ag**gatact |
| 2573 - 2613 | | 0.83 | | tcttcac**ag**agcccg | 2573 - 2613 | | | 0.83 | tcttcac**ag**agcccg |
| 2666 - 2706 | | 0.90 | | gctctac**ag**acaatc | 2841 - 2881 | | | 0.95 | tgtctgc**ag**agcttg |

<https://services.healthtech.dtu.dk/service.php?NetGene2-2.42>

********************** NetGene2 v. 2.42 ************************

The sequence: **wild** has the following composition:

Length: 2950 nucleotides.

25.2% A, 24.8% C, 22.7% G, 27.2% T, 0.0% X, 47.6% G+C

Donor splice sites, direct strand

---------------------------------

pos 5'->3' phase strand confidence 5' exon intron 3'

70 2 + 0.00 TGCACGGATG^GTAAGGAATA

145 0 + 0.71 ACCGACCCGG^GTGTGTACAC

1415 2 + 0.39 ATATGCTTAG^GTAATGACAG

1887 0 + 0.54 TATGTTCCAG^GTGGGTGTGG

1902 0 + 0.75 TGTGGAGGAG^GTGAGGGGAT

2771 0 + 0.99 GCTGGATGAG^GTAAGCCTGG H

Acceptor splice sites, direct strand

------------------------------------

pos 5'->3' phase strand confidence 5' ntron exon 3'

509 1 + 0.16 TATGTTCCAG^GCACTCCTCT

729 1 + 0.25 GTTCTTCCAG^GATTTTTCTA

1473 0 + 0.07 GATTAATGAG^TCTCCCGCCC

1491 0 + 0.67 CCCCTCCCAG^TCTATTCAGC

1500 0 + 0.31 GTCTATTCAG^CATGATCTGG

2296 1 + 0.77 TCTCTTCCAG^GAATAGTCAA

2381 1 + 0.29 TCCTTTGAAG^GATACTAAGG

2593 1 + 0.43 GTCTTCACAG^AGCCCGGAAA

2686 0 + 0.39 TGCTCTACAG^ACAATCATTC

********************** NetGene2 v. 2.42 ************************

The sequence: **mutant** has the following composition:

Length: 2946 nucleotides.

25.2% A, 24.8% C, 22.7% G, 27.3% T, 0.0% X, 47.6% G+C

Donor splice sites, direct strand

---------------------------------

pos 5'->3' phase strand confidence 5' exon intron 3'

70 2 + 0.00 TGCACGGATG^GTAAGGAATA

145 0 + 0.71 ACCGACCCGG^GTGTGTACAC

1415 2 + 0.39 ATATGCTTAG^GTAATGACAG

1887 0 + 0.54 TATGTTCCAG^GTGGGTGTGG

1902 0 + 0.75 TGTGGAGGAG^GTGAGGGGAT

2767 0 + 0.99 GCTGGATGAG^GTAAGCCTGG H

Acceptor splice sites, direct strand

------------------------------------

pos 5'->3' phase strand confidence 5' intron exon 3'

509 1 + 0.16 TATGTTCCAG^GCACTCCTCT

729 1 + 0.25 GTTCTTCCAG^GATTTTTCTA

1473 0 + 0.07 GATTAATGAG^TCTCCCGCCC

1491 0 + 0.67 CCCCTCCCAG^TCTATTCAGC

1500 0 + 0.31 GTCTATTCAG^CATGATCTGG

2296 1 + 0.77 TCTCTTCCAG^GAATAGTCAA

2381 1 + 0.29 TCCTTTGAAG^GATACTAAGG

2593 1 + 0.43 GTCTTCACAG^AGCCCGGAAA

2634 0 + 0.07 CTCCAAGCAG^CAGAACTTCT

### <https://www.fruitfly.org/cgi-bin/seq_tools/splice.pl>

### Acceptor site predictions for wild :

**Start End Score Intron Exon**
 489 529 0.79 tgagctcctactatgttccaggcactcctctagcaaacaaa
 566 606 0.68 agcaaggtctccctcttgtagagtttatattctagtatttt
 709 749 0.78 atctagtatatgttcttccaggatttttctatgcacacact
 783 823 0.84 attgtatgtacctctttgcagcctgcttttttcagttagtt
 797 837 0.69 tttgcagcctgcttttttcagttagttttttttgttttttt
 863 903 0.94 aagtcttgctctattccctaggctggagcacagtggtgcca
 937 977 0.65 aaactaattctcctgcctcagcctcccgacatagctgggat
 1005 1045 0.96 aatttttgtattttttagtagagacggggtttcaccatgtt
 1814 1854 0.71 gatctcctcgggctttcttagaattctctccctgggcactg
 1866 1906 0.84 ttcttgaatattatgttccaggtgggtgtggaggaggtgag
 2276 2316 0.99 ctcaacctctttctcttccaggaatagtcaaccctggatgg
 2361 2401 0.94 ctaccccttcctcctttgaaggatactaaggggtccagaaa
 2573 2613 0.83 gagtggtctgtgtcttcacagagcccggaaaatgaactagt
 2666 2706 0.90 tctctggttactgctctacagacaatcattccggttcagta

### Acceptor site predictions for mutant :

**Start End Score Intron Exon**
 489 529 0.79 tgagctcctactatgttccaggcactcctctagcaaacaaa
 566 606 0.68 agcaaggtctccctcttgtagagtttatattctagtatttt
 709 749 0.78 atctagtatatgttcttccaggatttttctatgcacacact
 783 823 0.84 attgtatgtacctctttgcagcctgcttttttcagttagtt
 797 837 0.69 tttgcagcctgcttttttcagttagttttttttgttttttt
 863 903 0.94 aagtcttgctctattccctaggctggagcacagtggtgcca
 937 977 0.65 aaactaattctcctgcctcagcctcccgacatagctgggat
 1005 1045 0.96 aatttttgtattttttagtagagacggggtttcaccatgtt
 1814 1854 0.71 gatctcctcgggctttcttagaattctctccctgggcactg
 1866 1906 0.84 ttcttgaatattatgttccaggtgggtgtggaggaggtgag
 2276 2316 0.99 ctcaacctctttctcttccaggaatagtcaaccctggatgg
 2361 2401 0.94 ctaccccttcctcctttgaaggatactaaggggtccagaaa
 2573 2613 0.83 gagtggtctgtgtcttcacagagcccggaaaatgaactagt
 2841 2881 0.95 gggccccttccctgtctgcagagcttgcctccaatcaggac
